# Tibial eminence fracture with midsubstance anterior cruciate ligament tear in a 10-year-old boy: A case report

**DOI:** 10.1016/j.ijscr.2019.12.036

**Published:** 2020-01-09

**Authors:** Shohei Yamauchi, Shizuka Sasaki, Yuka Kimura, Yuji Yamamoto, Eiichi Tsuda, Yasuyuki Ishibashi

**Affiliations:** a Department of Orthopaedic Surgery, Graduate School of Medicine, Hirosaki University, Japan; b Department of Rehabilitation Medicine, Graduate School of Medicine, Hirosaki University, Japan

**Keywords:** Anterior cruciate ligament, Tibial eminence fracture, Pediatric, Case report

## Abstract

**Introduction:**

There are few reports about tibial eminence fractures with a concomitant midsubstance ACL tear in children. In this report, we present a case of a tibial eminence fracture with an ACL midsubstance tear in a 10-year-old boy.

**Presentation of case:**

The boy twisted his right knee and was seen at our facility 8 days later. Magnetic resonance imaging revealed an osteochondral fragment in the intercondylar fossa and a high-intensity area in the ACL midsubstance. This injury was diagnosed as a type III tibial eminence fracture by Meyers and McKeever classification. On the 10^th^ day after the injury, the patient underwent arthroscopic reduction and fixation. During arthroscopy proximally displaced avulsion fragment in the intercondylar fossa was found. Although the continuity of the anteromedial bundle of the ACL was confirmed, the posterolateral bundle was completely torn and the tension of ACL was weakened. The fragment and torn ACL were fixed with pull out suture. One year after the operation, the patients can play sports without any pain.

**Discussion:**

This case demonstrates that a tibial eminence fracture with an ACL midsubstance tear can occur in a child. Despite the rarity of such cases, preoperative MRI studies are necessary to detect and diagnose them. MRI also allows surgeons to identify cases that might need ACLR.

**Conclusion:**

It should be noted that a midsubstance ACL tear can occur with a tibial eminence fracture even in skeletally immature patients, and that preoperative MRI is necessary to diagnose and select the appropriate treatment.

## Highlights

• We present a tibial eminence fracture with an ACL tear in a 10-year-old boy.
• A tibial eminence fracture with an ACL midsubstance tear can occur in a child.
• Preoperative MRI studies are necessary to detect and diagnose them.
• MRI also allows surgeons to identify cases that might need ACLR.

## 1 Introduction

Tibial eminence fractures, which are avulsion fractures of the attachment of the anterior cruciate ligament (ACL) [1], are most common in children and adolescents from 8 to 14 years of age [2], with an incidence of 3 per 10,000 children per year [3]. Without appropriate diagnosis and treatment, a tibial eminence fracture can damage ACL function or restrict the range of motion of the knee. Tibial eminence fractures with a concomitant midsubstance ACL tear have been reported in adults [[4], [5], [6]]; however, there are few reports of such cases in children. We here report a case of a tibial eminence fracture with a midsubstance tear of the posterolateral bundle (PLB) in a 10-year-old boy. This report was made in line with SCARE criteria [7].

## 2 Case report

**Fig. 1:**
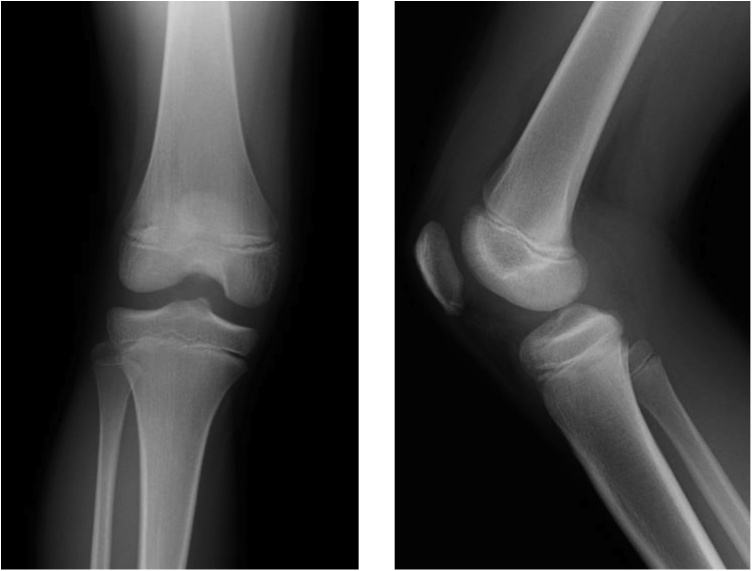
Radiographs did not show clear evidence of a tibial eminence fracture.

**Fig. 2:**
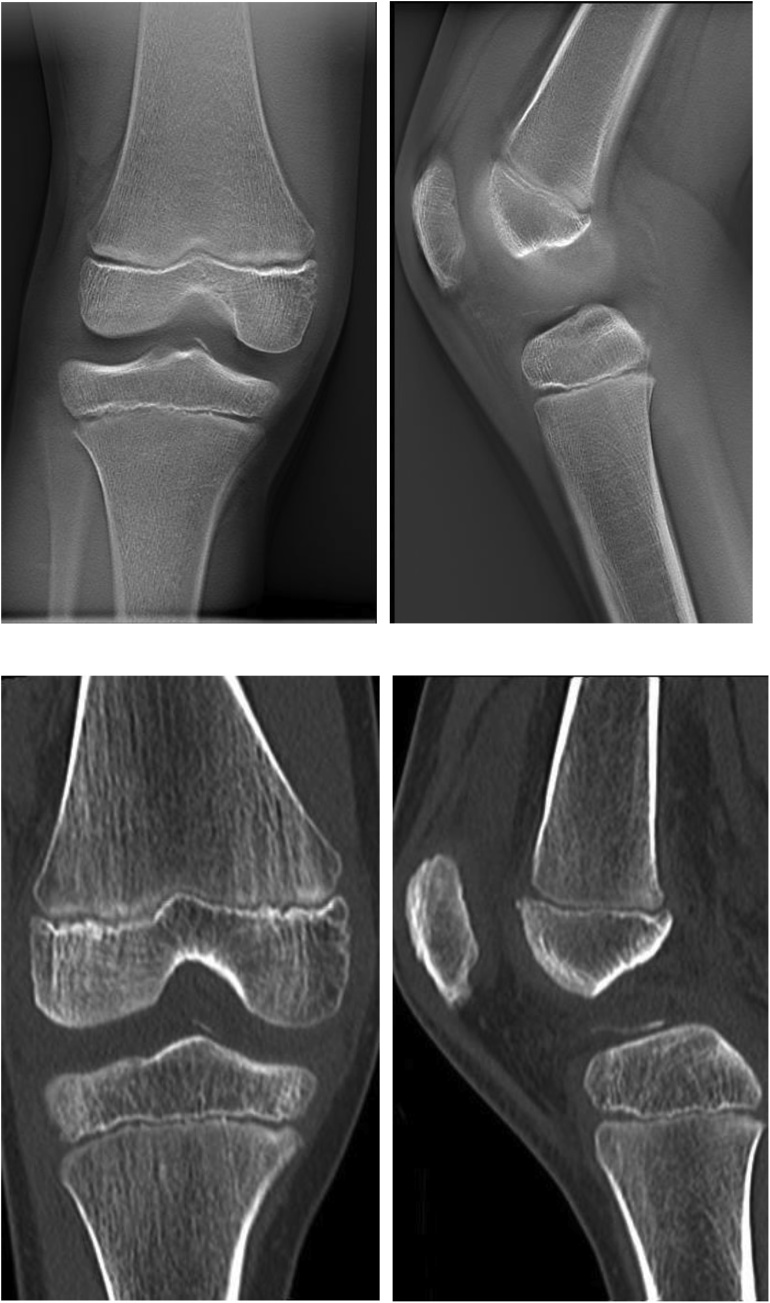
Computerized tomography clearly showed a tibial eminence fracture and an avulsion fragment in the intercondylar fossa.

**Fig. 3:**
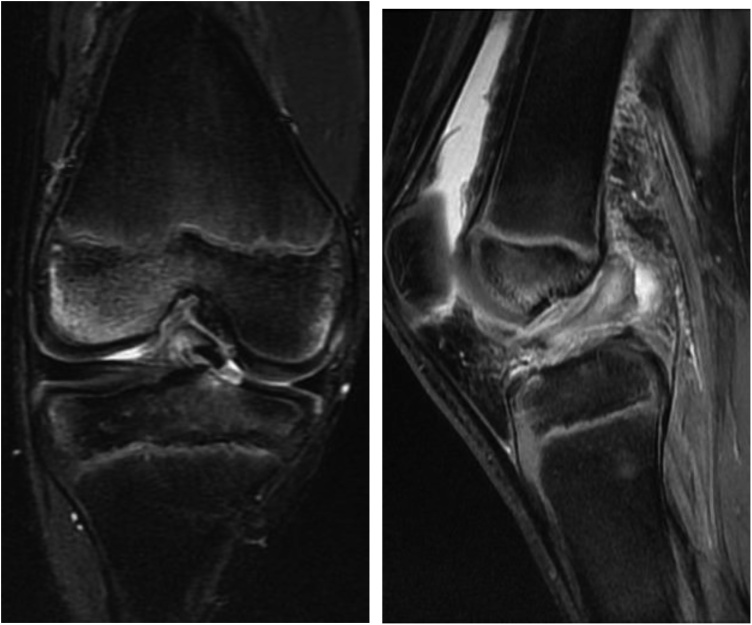
Magnetic resonance imaging revealed an osteochondral fragment in the intercondylar fossa and a signal change in the anterior cruciate ligament midsubstance.

The boy fell and twisted his right knee while skiing, and was seen at our facility 8 days later. The right knee had a large effusion, its range of motion was restricted, and pain in the knee was too severe to perform anterior drawer or pivot shift tests. The result of Lachman test was positive. Anterior tibial translation, measured with a KT-1000™ arthrometer, was 10 mm in the right knee and 7 mm in the left. Radiographs of the right knee revealed an open physis of the femur and tibia but fracture line was barely visible (Fig. 1). Tomosynthesis and computerized tomography (Fig. 2) confirmed an avulsion fragment of the tibial eminence. Magnetic resonance imaging (MRI) revealed an osteochondral fragment in the intercondylar fossa and a high-intensity area in the ACL midsubstance on sagittal T2-weighted images (Fig. 3). Open physis of the femur and tibia was also confirmed. The injury was diagnosed as a type III tibial eminence fracture by Meyers and McKeever classification [8].

**Fig. 4:**
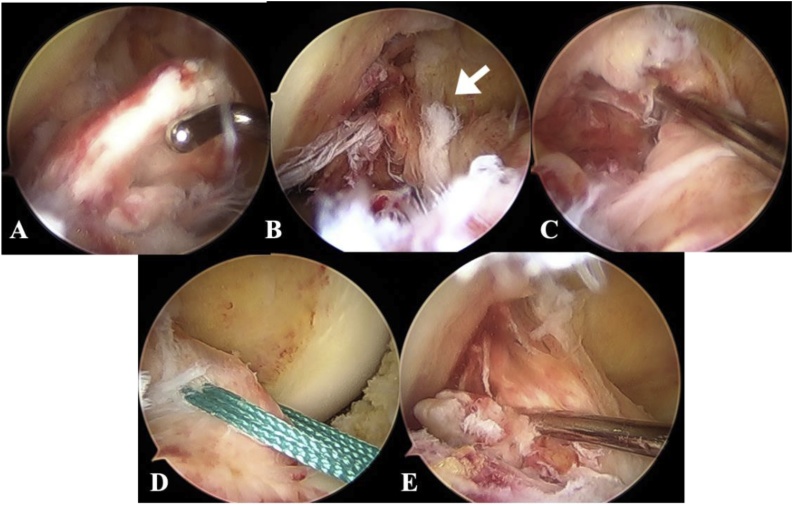
Arthroscopy revealed A) an avulsion fragment in the intercondylar fossa, B) anteromedial bundle continuity (arrow), and C) a torn posterolateral bundle and weakened ACL tension. D) We performed pull-out suture fixation, and E) confirmed tension on the anteromedial bundle.

On the 10^th^ day after the injury, the patient underwent arthroscopic reduction and fixation; we had prepared for physeal sparing ACL reconstruction (ACLR) if he had an ACL midsubstance tear. During arthroscopy we found a proximally displaced avulsion fragment in the intercondylar fossa (Fig. 4A), We also confirmed the continuity of the anteromedial bundle (AMB) of the ACL (Fig. 4B) but the PLB was completely torn (Fig. 4C). The tibial part of the ACL midsubstance was sutured using a Knee Scorpion (Arthrex, Naples, FL, USA) and two no. 2 Ethibond Excel sutures (Ethicon Inc, Somerville, New Jersey, USA).

**Fig. 5:**
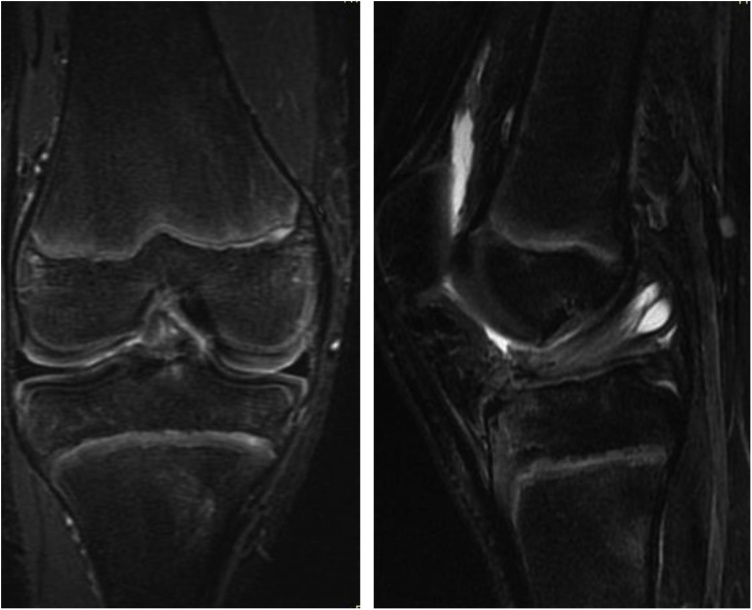
Magnetic resonance imaging obtained 4 months after surgery showed decreased signal intensity around the tibial eminence. Although the signal intensity of ACL fiber was still high, the continuous ACL was confirmed.

Two bone tunnels were made just medial and lateral to the fracture site using a 2.4-mm guide wire, and the two no. 2 Ethibond Excel sutures were pulled out (Fig. 4D). ACL tension was confirmed by probing (Fig. 4E), and the knee was stable after fixation of the bone fragments under anesthesia. The knee was immobilized in a cylinder cast for 3 weeks. The patient began range-of-motion and partial weight-bearing exercises as soon as the cast was removed, and was able to start placing his full weight on the knee 5 weeks after the operation. MRI studies conducted 4 months after surgery showed a decreased signal intensity around the tibial eminence. Although the signal intensity of ACL fiber was still high, the continuity of the ACL was confirmed (Fig. 5).

**Fig. 6:**
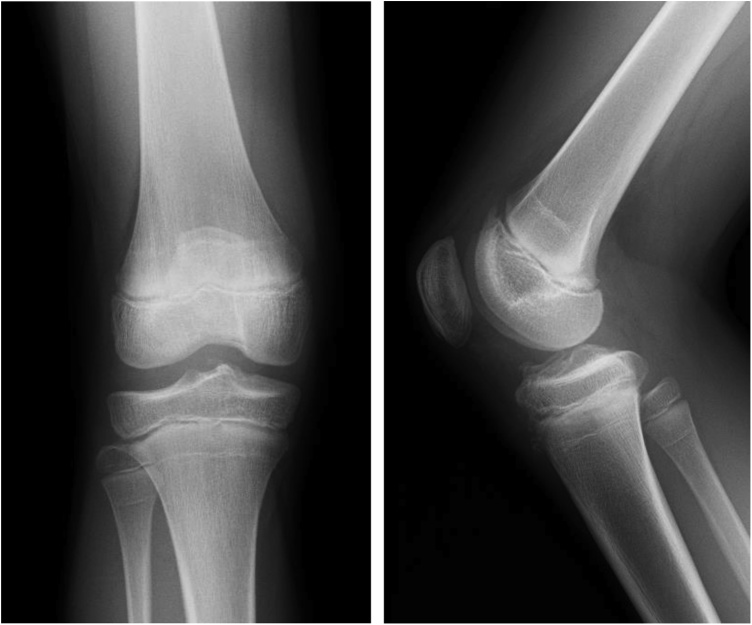
Radiographs showed bone healing and no dislocation of the bone fragment in the knee at the 1-year follow-up.

One year after the operation, range of motion of the right knee showed 0° extension and 150° flexion, with a 1-cm heel height difference (HHD). The HHD has been used to detect subtle knee flexion contractures. The Lachman, anterior drawer, and pivot shift tests were all negative. The side-to-side difference in anterior tibial translation, measured with the KT-1000™, was +1 mm (8 mm right knee and 7 mm left). The patient was able to swim, ski, and play baseball without any pain, sensation of instability, or effusion. Radiographs showed bone healing and no displacement of the bone fragment (Fig. 6). The patient’s family consented to the submission of this case data and report for publication.

## 3 Discussion

This case demonstrates that a tibial eminence fracture with an ACL midsubstance tear can occur in a child. In studies of associated injuries in tibial eminence fractures, a concomitant ACL midsubstance tear was found in 3 of 11 adults (27.3%) but in none of 19 cases of acute tibial eminence fractures in children [4]. Griffith et al. [9] reported a case of a tibial eminence fracture with an ACL midsubstance tear in a 15-year-old boy, but did not describe the treatment or clinical result. The rarity of pediatric cases of tibial eminence fractures with an ACL midsubstance tear makes it difficult to establish treatment strategies and clarify long-term clinical outcomes for these patients.

Adam et al. [10] investigated associated knee injuries in 20 pediatric cases of tibial eminence fracture, and found 6 meniscus tears and 2 ligamentous injuries (a posterolateral corner injury and a medial collateral ligament tear). They concluded that for cases that will be treated arthroscopically, preoperative MRI does not provide any additional information since associated ligamentous injuries are detected much less often than meniscus injuries. However, our case demonstrates that an ACL midsection tear can accompany a tibial eminence fracture even in children. Despite the rarity of such cases, preoperative MRI studies are necessary to detect and diagnose them. MRI also allows surgeons to identify cases that might need ACLR and to prepare accordingly. Chotel et al. [11] recently described a case of cartilaginous tibial eminence fracture (CTEF) as a new pattern of ACL tear in children under 9 years of age. CTEF is difficult to diagnose because it involves purely cartilaginous avulsions of the ACL insertion on either the femoral or tibial side, and traditional findings after ACL injury are absent on MRI, although a fluid signal underneath the cartilaginous fragment reaching up to the ossified epiphysis, or a double PCL sign, may point doctors toward the appropriate diagnosis [12]. In our case, coronal MRI confirmed the presence of small bone fragments and a cartilaginous avulsion, which is consistent with CTEF (Fig. 3). Thus, MRI is helpful for diagnosing ligamentous injuries in CTEF, as in our present case. CTEF is one of the injuries that can be misdiagnosed, and orthopedic surgeons need to pay attention to this fracture.

By Meyers and MacKeever classification, conservative therapy is considered appropriate for typeⅠor type II tibial eminence fractures. Arthroscopic-assisted internal fixation is considered appropriate for type III and IV fractures, and for type II fractures when closed reduction is impossible [4]. We performed arthroscopic reduction and fixation while preparing physeal sparing ACLR because this case was a type Ⅲ tibial eminence fracture and midsubstance ACL tear was confirmed in preoperative MRI. In fact, we confirmed the midsubstance tear of the PL bundle in arthroscopic findings.

The treatment of midsubstance ACL tear in childhood is controversial. Nonoperative treatment may not sufficiently prevent knee instability and can lead to meniscal and cartilage damage due to recurrent instability episodes [[13], [14], [15]]. Operative treatment, on the other hand, may cause growth disturbances of the involved leg due to involvement of the physis [16]. As operative treatments, transphyseal ACLR or physeal sparing ACLR are typically reported. A systematic review identified that three-fourths of angular deformity cases and one-half of limb length discrepancy cases following pediatric ACL reconstruction occur after transphyseal ACLR, with the remainder occurring after physeal-sparing ACLR in open physis children [17]. Whichever surgical method we use, complications cannot be completely avoided. It is important to educate the patient’s parents and obtain consent since there is a risk of a valgus deformity or a disturbance of growth along the length of the operated leg after ACLR [18,19]. First of all, we follow up carefully with rehabilitation and restriction of physical activity for these cases in our institution. If the drop-out sign is observed in MRI [20], we perform transphyseal ACLR. However, if serious meniscus injury is confirmed during the preoperative period, physeal sparing ACLR may be considered for open physis patients. In this case, we did not conduct ACLR because stability was obtained after bone-fragment fixation.

Han et al. [21] described a case of tibial eminence fracture with a midsubstance ACL tear in a 13-year-old boy; in that case, the AMB was torn and the partial continuity of the PLB on an osteochondral fragment was confirmed by arthroscopy. The osteochondral fragment was fixed by pull-out suture. However, knee laxity was evident at the 6-month follow-up, and they performed ACLR after closing the epiphyseal line of the femur and tibia. Their case suggested that the possibility that knee instability may occur after osteochondral fragment fixation in the cases with an ACL midsubstance tear. Careful long-term follow-up is particularly important in pediatric cases.

## 4 Conclusion

In conclusion, it should be noted that a midsubstance ACL tear can occur with a tibial eminence fracture even in skeletally immature patients, and that preoperative MRI is necessary to diagnose this type of fracture and select appropriate treatment. If MRI shows the possibility of a midsubstance tear, the surgeon should prepare for ACLR and get the parent’s informed consent before surgery.

## Sources of funding

No.

## Ethical approval

Since our article is a case report, no approval from the Ethics Committee is required in our institution.

## Consent

We confirm parental consent on behalf of the patient.

## Author contribution

Shizuka SASAKI. M.D. Ph.D. writing the paper.

Yuka KIMURA. M.D. Ph.D. writing the paper.

Yuji YAMAMOTO. M.D. Ph.D. study concept.

Eiichi TSUDA. M.D. Ph.D. study concept.

Yasuyuki ISHIBASHI. M.D. Ph.D. study concept.

## Registration of research studies

Research registry 5093.

## Guarantor

Shohei YAMAUCHI. M.D.

## Provenance and peer review

Editorially reviewed, not externally peer-reviewed.

## Declaration of Competing Interest

No.
